# Elevated Expression of Gamma-Glutamyl Hydrolase Is Associated With Poor Prognosis and Altered Immune Signature in Uterine Corpus Endometrial Carcinoma

**DOI:** 10.3389/fgene.2021.764194

**Published:** 2022-01-10

**Authors:** Cong Yu, Haining Qi, Yanhui Zhang, Wen Zhao, Guoying Wu

**Affiliations:** ^1^ School of Life Sciences, Qilu Normal University, Jinan, China; ^2^ Department of Obstetrics, Affiliated Hospital of Shandong University of Traditional Chinese Medicine, Jinan, China; ^3^ Maternal and Child Health Care Hospital of Shandong Province, Shandong University, Jinan, China; ^4^ College of Life Sciences, Shandong Normal University, Jinan, China

**Keywords:** GGH, prognosis, uterine corpus endometrial carcinoma, TCGA, immune infiltration

## Abstract

Uterine corpus endometrial carcinoma (UCEC) is a common malignant tumor of the female reproductive system with poor prognosis in advanced, recurrent, and metastatic cases. Identification of reliable molecular markers will help in the development of clinical strategies for early detection, diagnosis, and intervention. Gamma-glutamyl hydrolase (GGH) is a key enzyme in folate metabolism pathway. High expression of GGH is associated with severe clinicopathological features and poor prognosis of several cancers. High GGH expression is also related to cell resistance to antifolate drugs such as methotrexate. In this study we focused on the prognostic value of immunohistochemical GGH expression level in UCEC tissue and RNA-seq data from The Cancer Genome Atlas to establish associations with clinical features and outcomes. Further, we conducted comprehensive bioinformatics analyses to identify and functionally annotate differentially expressed genes (DEGs) associated with UCEC upregulation and assessed the effects of upregulation on immune infiltration. Both GGH mRNA and protein expression levels were elevated in tumor tissues, and higher expression was significantly associated with advanced clinicopathological features and poor prognosis by univariate analysis. Further multivariate analysis identified elevated GGH expression as an independent risk factor for poor outcome. Nomograms including GGH expression yielded a c-index for disease-specific survival prediction of 0.884 (95% confidence interval: 0.861–0.907). A total of 520 DEGs (111 upregulated and 409 downregulated) were identified between high and low GGH expression groups. Analysis using Gene ontology, Kyoto Encyclopedia of Genes and Genomes pathway, Gene set enrichment analysis, and protein‒protein interaction indicated significant associations of altered GGH expression with cell proliferation, immune response, and the occurrence and development of UCEC tumors. Finally, GGH expression level was associated with high Th2 cell and low natural killer CD56bright cell infiltration. Collectively, these findings indicate that GGH drives UCEC progression and could be a useful biomarker for survival prediction as well as a therapeutic target.

## Introduction

Uterine corpus endometrial carcinoma (UCEC) is one of the three most common malignant tumors in gynecology (Bray et al., 2018). The Chinese National Cancer Center reported an incidence of 63.4/100,000 and mortality of 21.8/100,000 in 2015 (Chen et al., 2016), and both indices continue to rise domestically and globally. In the early stage of UCEC, the tumor is limited to the uterus and prognosis is good even with surgical resection only. Patients with advanced UCEC or recurrence can still benefit from adjuvant therapies, such as chemotherapy, radiotherapy, or endocrine therapy (Morice et al., 2016; McEachron et al., 2020). However, for advanced patients and young patients with fertility requirements, as well as patients with diabetes or other diseases, the efficacy of existing treatments is limited and prognosis is poor (Barcellini et al., 2020). Therefore, early detection and treatment of UCEC is essential, and biomarkers with high accuracy, reliability, and sensitivity could greatly improve detection and prognosis. Although several biomarkers and therapeutic targets of UCEC have been reported, such as TPX2, PIK3CA, and ACE2, the current array is insufficient for routine early detection and effective treatment in more advanced cases (Jiang et al., 2018; Pan et al., 2019; Urick and Bell, 2019; Yang et al., 2020).

Folate metabolism plays an essential role in DNA synthesis, methylation, cell proliferation, and cell repair. Enzymes involved in folate metabolism are reported to be abnormal in the highly proliferating cancerous cells. Gamma-glutamyl hydrolase (GGH) is a key enzyme in maintaining intracellular folate homeostasis. It catalyzes the hydrolysis of polyglutamylated folate into monoglutamylated folate, which is subsequently exported from the cell (Shane, 2001; Ducker and Rabinowitz, 2017). The expression level of GGH strongly influences global DNA methylation status, DNA methyltransferase activity, promoter DNA methylation, and gene expression (Kim et al., 2013; Wang et al., 2014; Kim et al., 2015). Elevated GGH expression was found in breast, ERG-negative prostate, gallbladder, and gastric cancers compared to matched noncancerous tissues (Shubbar et al., 2013; Odin et al., 2019; Zali et al., 2019; Maezawa et al., 2020). High levels of GGH mRNA expression are significantly correlated with more advanced histological type, vascular invasion, and poor survival rate compared to low GGH expression levels in cervical cancer, gallbladder cancer, and breast cancer (Shubbar et al., 2013; Odin et al., 2019; Peng et al., 2019; Zali et al., 2019; Maezawa et al., 2020). Studies also showed that low GGH expression in cells can increase the chemosensitivity of cancer cells to antifolate drugs such as methotrexate, whereas high GGH expression is related to cell resistance to antifolates (Rhee et al., 1993). The relative expression status and the role of GGH in gynecological tumors such as UCEC are still unclear and require further research.

In this study, UCEC RNA-seq data from The Cancer Genome Atlas (TCGA) were used to examine whether GGH expression is also elevated in UCEC tumors. The expression level of GGH in UCEC was confirmed by immunostaining of patient tissue samples. We also evaluated the association between GGH expression and various clinicopathological as well as outcome indicators, then constructed nomograms to evaluate prognostic efficacy. Differential gene expression between the high and low GGH expression groups was analyzed to identify potential downstream and upstream pathways regulating tumor progression and outcome. Finally, we examined the correlation between GGH expression and immune infiltration. Our results suggest that high GGH expression drives UCEC progression, possibly by disrupting molecular pathways regulating the cell cycle, apoptosis, and immune responses. Elevated GGH expression also predicted outcome with high accuracy, suggesting its utility as a prognostic marker and potential therapeutic target for UCEC.

## Materials and Methods

### Immunohistochemical Staining

Tissue microarray (TMA) (Cat No. OD-CT-RpUtr03-002) paraffin blocks of UCEC tissues were purchased from Shanghai Outdo Biotech Company (Shanghai, China). A total of 31 pairs of cancerous and paracancerous tissue samples were subjected to IHC staining. Each TMA slide was first stained with a rabbit anti-GGH antibody (dilution, 1:200; ABP56886; Abbkine, Wuhan, China) and then incubated with horseradish peroxidase-conjugated anti-rabbit IgG secondary antibody (dilution, 1:200; GB23303; Servicebio, Inc., Woburn, MA). After rinsing, color was developed using 3, 3′-diaminobenzidine (DAB, Servicebio, Inc.). Sections were counterstained with hematoxylin and photographed at 400× magnification using an XSP-C204 microscope (COIC, Chongqing, China). Images were then captured using Pannoramic viewer (3DHISTECH Kft; Budapest, Hungary) and analyzed using Quant Center (3DHISTECH). Immunohistochemistry score (H-SCORE) was calculated as H-SCORE = ∑ (PI × I) = (percentage of cells with weak intensity × 1) + (percentage of cells with moderate intensity × 2) + (percentage of cells with strong intensity × 3), where PI is the proportion of positive cells among all cells in the section and I is the coloration intensity (Azim et al., 2015; Yeo et al., 2015).

### Data Source and Preprocessing

The RNA-seq data of level-3 HTseq-FPKM and accompanying patient-specific clinical information for multiple UCEC projects were downloaded from TCGA (https://prtal.gdc.cancer.gov/), and RNA-seq data of transcripts per million (TPM) reads from TCGA and GTExPortal, with unified processing using the TCGA Toil application, were download from UCSC XENA (https://xenabrowser.net/datapages/) (Vivian et al., 2017). In total, 543 cases with clinical information were collected after discarding those with overall survival of less than 30 days. The RNA-seq data of level 3 HTseq-FPKM were converted into TPM format for subsequent analysis. Unavailable or unknown clinical parameters were considered as missing values. The study was conducted in accordance with the publication guidelines stated by TCGA (http://cancergenome.nih.gov/publications/publicationguidelines).

### Construction and Evaluation of Nomograms

According to the Cox multifactor regression model, nomograms were constructed using the rms package (Version: 5.1-3; https://cran.r-project.org/web/package/rms/index.html) to identify independent prognostic factors. A concordance index (c-index) was calculated using a bootstrap approach with 1000 resamples to determine the discrimination power of the nomogram (Iasonos et al., 2008). Calibration plots were then constructed to evaluate the predictive accuracy of the nomogram according to the consistency between predicted and actual overall survival (OS), disease-specific survival (DSS), and disease-free survival at 1, 3, and 5 years.

### Analysis of Differentially Expressed Genes Between High and Low GGH Expression Subgroups

Tumor samples were divided into high and low expression subgroups according to the median GGH expression. DEGs were identified from HTSeq-Counts using DESeq2 software (Love et al., 2014) with thresholds of |log2 fold change (logFC)| > 2 and adjusted *p* < 0.01. Results of DEG analysis are displayed as volcano plots and heat maps.

### Functional and Pathways Enrichment Analysis

Gene ontology (GO) classification as “biological process” (BP), “cellular components” (CC), or “molecular function” (MF), and Kyoto Encyclopedia of Genes and Genomes (KEGG) pathway enrichment analysis of DEGs between high and low GGH expression groups were performed (Yu et al., 2012). Terms with *p* < 0.05 after adjustment using the Benjamini–Hochberg method were considered significant. Gene set enrichment analysis (GSEA) between high and low GGH expression groups was performed (Subramanian et al., 2005; Yu et al., 2012), and gene set permutations were performed 1,000 times per analysis. The expression level of GGH was used as a phenotype label and enriched pathways were identified according to |NES| > 1, adjusted *p* < 0.05, and false discovery rate q-value < 0.25. The enrichment analyses all above were performed by the R package ClusterProfiles (3.14.3).

### Construction of Protein‒Protein Interaction Networks

The Search Tool for Retrieval of Interacting Genes (STRING, http://string-db.org/) database was used to analyze PPI networks among DEGs (Szklarczyk et al., 2019). An interaction score >0.4 was used as the cutoff to assess the potential PPI network relationship. Cytoscape software (version 3.7.0) was used to visualize the PPI network, and Cytohubba was used to identify densely connected network components and to extract the top 10 hub genes (Doncheva et al., 2019).

### Immune Infiltration Analysis

Immune infiltration analysis was performed for 24 distinct immune cell types within tumor samples by the single sample GSEA (ssGSEA) method using GSVA software (http://www.bioconductor.org/packages/release/bioc/html/GSVA.html) in the R environment. Based on the characteristic genes of these 24 immune cell types (Bindea et al., 2013), relative enrichment scores were calculated for each tumor sample. Spearman correlation analysis was used to assess the associations between GGH expression and infiltration of each immune cell type. The Wilcoxon rank sum test was used to compare cell immune infiltration between high and low GGH expression groups.

### Statistical Analysis

All statistical analyses and plotting were conducted using the R program environment (v.3.6.2). The Wilcoxon rank sum and signed rank tests were used to compare GGH expression between tumor samples with paired or unpaired control samples. The Kruskal–Wallis rank sum test and logistic regression were used to analyze the correlations between the clinicopathological features and GGH expression, whereas Pearson’s χ^2^ test, Fisher’s exact test, and Wilcoxon rank sum test were used to analyze the direct correlation between clinicopathological variables and GGH expression level (high or low according to the median). Receiver operating characteristic (ROC) analysis was performed using the pROC package (Robin et al., 2011) to evaluate the effectiveness of GGH expression level (high or low) for discriminating UCEC samples from control samples. Kaplan‒Meier curves were constructed using the survminer package (Version 0.4.4; https://CRAN.R-project.org/package=survminer) to evaluate the utility of GGH for predicting OS, DDS, and the progression-free interval (PFI). Survival differences between high and low expression groups were examined by the log-rank test. Univariate and multivariate Cox regression analyses were used to identify independent prognostic factors related to survival. Variables with *p* < 0.1 by univariate Cox regression analysis were incorporated into the multivariate Cox regression model. Hazard ratios (HR) with 95% confidence interval (CI) were calculated to estimate the hazard risk of individual factors. Forest plots and Kaplan‒Meier curves were used for analysis of GGH prognostic efficacy within clinical subgroups stratified by parameters deemed significant by the multivariate Cox model. All tests were two-sided and *p* < 0.05 was considered statistically significant.

## Results

### Elevated GGH Protein and mRNA Expression in UCEC

**FIGURE 1:**
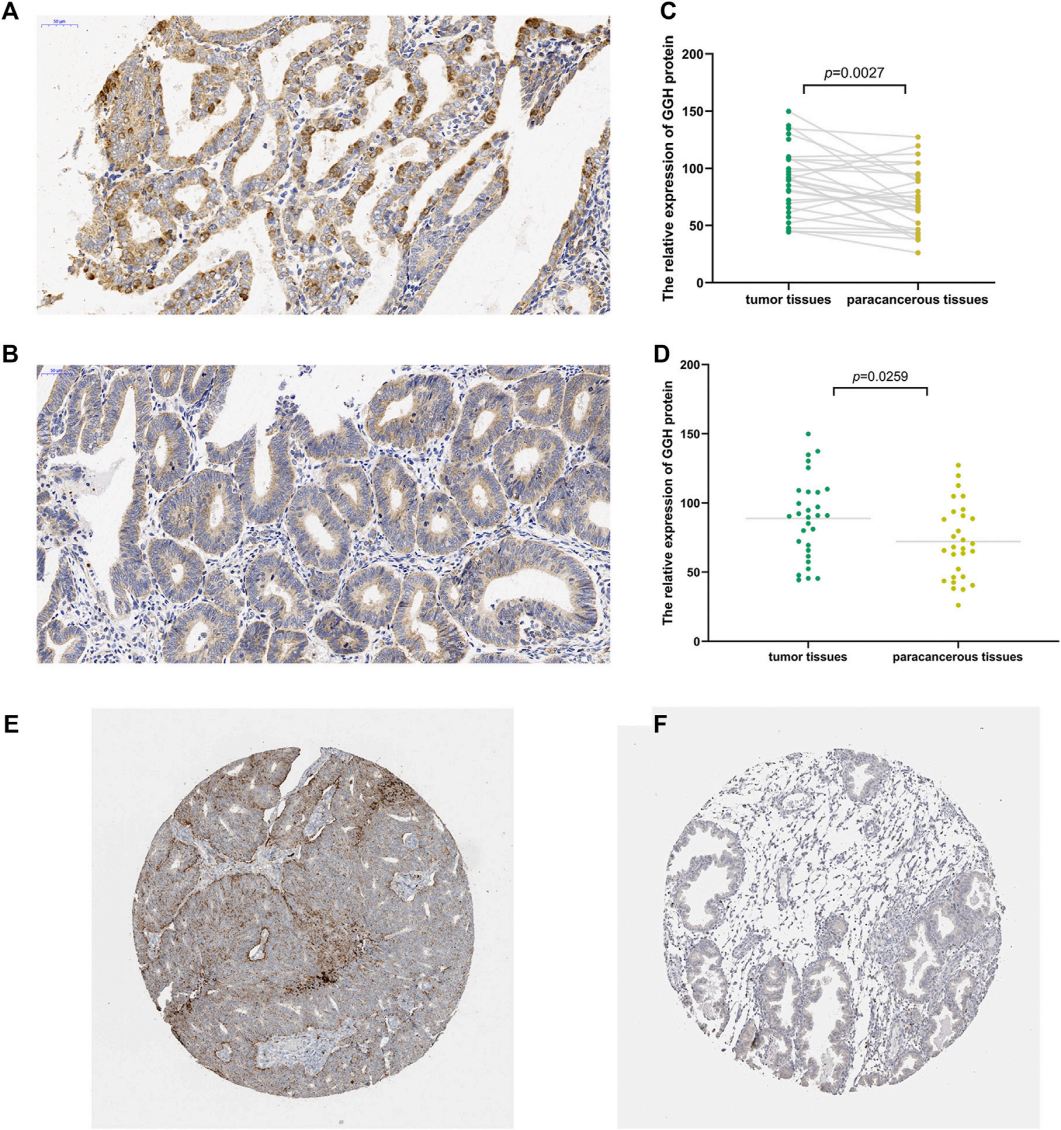
Elevated expression of GGH protein in UCEC tumor tissue. **(A,B)** Immunoexpression of GGH protein in UCEC tumor tissues and paired paracancerous tissue (× 200 magnification). **(C,D)** Comparisons of relative GGH protein expression in tumor tissues versus both paired and unpaired paracancerous tissues using H-scores. **(E,F)** Immunoexpression of GGH in UCEC tumor tissues and normal tissues from the Human Protein Atlas (×2 magnification).

**FIGURE 2:**
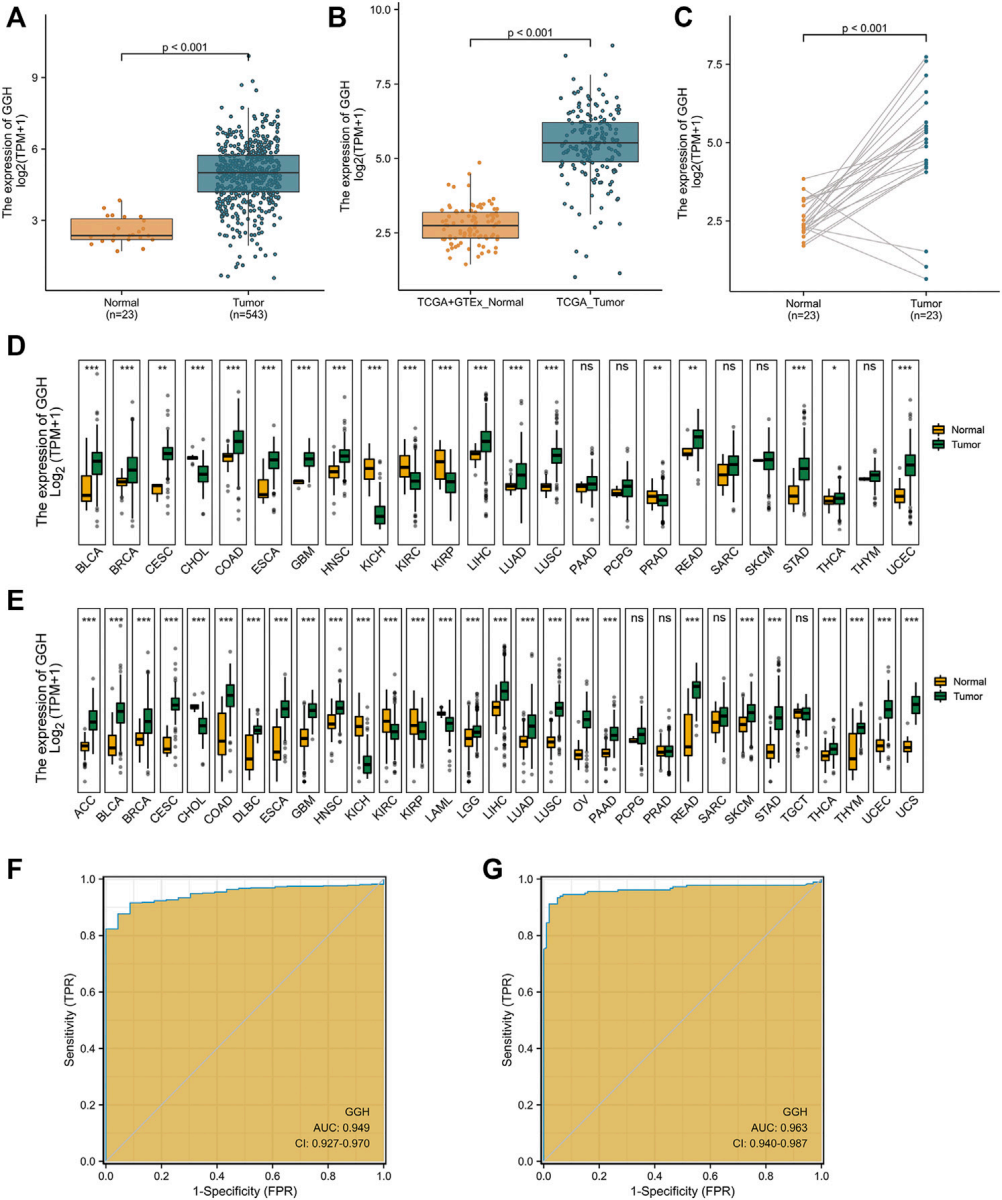
Elevated GGH mRNA expression in UCEC. **(A)** Elevated GGH mRNA in UCEC tumor samples compared to adjacent normal samples from TCGA. **(B)** Elevated GGH in UCEC tumor samples and normal samples from combined TCGA and GTEx datasets. **(C)** Differences in GGH mRNA expression between individual UCEC tumor samples and paired adjacent normal tissue samples from TCGA. **(D)** Differences in GGH mRNA expression between tumor tissues and paracancerous samples for 33 cancer types from TCGA. **(E)** Differences in GGH mRNA expression between tumor tissues and normal samples for 33 cancer types from the combined TCGA and GTEx dataset; ns: *p* ≥ 0.05; *: *p* < 0.05; **: *p* < 0.01; ***: *p* < 0.001. **(F,G)** ROC curve assessing the efficiency of GGH mRNA expression for distinguishing UCEC tumor tissues from nontumor tissues from the combined TCGA and GTEx dataset (AUC = 0.949) and TCGA only (AUC = 0.963). The abscissa is the False Positive Rate and the ordinate is the True Positive Rate.

To study the potential pathogenic functions of GGH in UCEC, we first compared GGH expression between tumor tissues and paracancerous tissues from 31 UCEC patients by immunohistochemistry. Denser immunostaining for GGH was observed in the cytoplasm of tumor cells (Figures 1A,B), and H-scores indicated significantly higher immunoexpression in tumor tissues compared to paired paracancerous tissues (Figure 1C, *p* = 0.0027) and unpaired control tissues (Figure 1D, *p* = 0.0259). Immunohistochemical results from the Human Protein Atlas also showed higher GGH expression in UCEC tumor tissues compared to normal tissues (Figures 1E,F). Again, immunoexpression was mainly in cytoplasm, consistent with our IHC staining results. Mean GGH mRNA expression was also higher in UCEC tumor tissues compared to adjacent normal tissues from TCGA database and in UCEC tissues versus normal tissues from the combined GTEx and TCGA dataset (both *p* < 0.001, Figures 2A,B). Moreover, GGH mRNA expression was elevated in 23 of the 27 individual UCEC tissue samples compared to adjacent tissues from TCGA (*p* < 0.001, Figure 2C).

We also conducted pancancer analysis to confirm our methodology against cancer types with known GGH elevation and to compare the magnitude of elevation in UCEC. Expression was significantly elevated in bladder urothelial carcinoma, breast invasive carcinoma, cervical squamous cell carcinoma and adenocarcinoma, cholangiocarcinoma, colon adenocarcinoma, esophageal carcinoma, glioblastoma multiforme, head and neck squamous cell carcinoma, kidney chromophobe, kidney renal clear cell carcinoma, kidney renal papillary cell carcinoma, liver hepatocellular carcinoma, lung adenocarcinoma, lung squamous cell carcinoma, rectum adenocarcinoma, stomach adenocarcinoma, and thyroid carcinoma according to the TCGA dataset (Figure 2D). Further, GGH expression was also elevated in adrenocortical carcinoma, diffuse large B cell lymphoma, acute myeloid leukemia, brain lower grade glioma, ovarian serous cystadenocarcinoma, and uterine carcinosarcoma, as evidenced by comparison to normal tissues from the combined GTEx and TCGA dataset (Figure 2E). This ubiquity of GGH overexpression in cancer suggests important contributions to tumorigenesis and (or) progression, and the relative elevation appeared higher in UCEC than many other tumor types.

We then performed ROC analysis to measure the capacity of GGH expression level to distinguish UCEC tumor tissues from nontumor tissues. The area under the curve (AUC) was 0.949 using combined TCGA and GTEx data and 0.963 using only TCGA data (Figures 2F,G), yielding specificity estimates of 95.7 and 98.0% and sensitivity estimates of 87.9 and 91.2%, respectively. We used the datasets in GEO to verify the ability of GGH expression levels to distinguish tumor tissues from non-tumor tissues, and the AUC was 0.711 (Supplementary Figure S1A). In addition, we further checked the ROC curves of patients with different histologic grades and verified them with independent datasets in GEO (Supplementary Figures S1B–G). These results suggested that GGH expression could distinguish patients with different clinical characteristics.

### Associations of GGH Expression Level With Clinicopathologic Variables

To identify associations between clinical parameters and GGH expression level, we first compared the numbers of high and low expression patients (272 cases of low and 271 cases of high expression in total) from TCGA stratified by various clinical classifications (Table 1). The proportions of low and high GGH expression cases differed significantly according to the clinical stage, histological grade, primary therapy outcome, race/ethnicity, surgical approach, and TP53 mutation status, but not by residual tumor percentage class, histological type, diabetes status, menopause status, hormone therapy treatment, or radiotherapy treatment. Further, weight and body mass index (BMI) differed significantly between high and low GGH expression groups while height did not.

**TABLE 1:** Associations between GGH expression level and clinicopathological features of UCEC.

Characteristics	GGH expression		*P*
	Low (*n* = 272)	High (*n* = 271)	
Clinical stage (%)			
Stage I	190 (69.9%)	149 (55.0%)	0.003^a^
Stage II	17 (6.2%)	34 (12.5%)	
Stage III	53 (19.5%)	71 (26.2%)	
Stage IV	12 (4.4%)	17 (6.3%)	
Histologic grade (%)			
G1	71 (26.8%)	27 (10.1%)	<0.001^a^
G2	72 (27.2%)	48 (18.0%)	
G3	122 (46.0%)	192 (71.9%)	
Residual tumor (%)			
R0	192 (91.9%)	180 (89.6%)	0.54
R1	11 (5.3%)	11 (5.5%)	
R2	6 (2.9%)	10 (5.0%)	
Primary therapy outcome (%)			
CR	236 (94.0%)	200 (89.7%)	0.035^a^ ^,^ ^b^
PD	6 (2.4%)	14 (6.3%)	
PR	4 (1.6%)	8 (3.6%)	
SD	5 (2.0%)	1 (0.4%)	
Histological type (%)			
Endometrioid	216 (79.4%)	191 (70.5%)	0.056
Mixed	9 (3.3%)	13 (4.8%)	
Serous	47 (17.3%)	67 (24.7%)	
Diabetes (%)			
No	163 (72.8%)	158 (72.5%)	1
Yes	61 (27.2%)	60 (27.5%)	
Menopause status (%)			
Peri	11 (4.5%)	6 (2.4%)	0.435
Post	218 (88.3%)	227 (90.8%)	
Pre	18 (7.3%)	17 (6.8%)	
Race (%)			
Asian	11 (4.4%)	9 (3.6%)	0.028^a^
Black or African American	41 (16.4%)	65 (26.2%)	
White	198 (79.2%)	174 (70.2%)	
Surgical approach (%)			
Minimally Invasive	85 (32.1%)	116 (45.3%)	0.003^a^
open	180 (67.9%)	140 (54.7%)	
Hormones therapy (%)			
No	150 (86.7%)	144 (86.7%)	1
Yes	23 (13.3%)	22 (13.3%)	
Radiation therapy (%)			
No	141 (53.6%)	133 (52.2%)	0.807
Yes	122 (46.4%)	122 (47.8%)	
TP53 status (%)			
Mutant	77 (28.9%)	112 (43.1%)	0.001^a^
Wild-type	189 (71.1%)	148 (56.9%)	
Age [median (IQR)]	63.00 [56.00, 71.00]	64.00 [57.00,72.00]	0.114^c^
Height [median (IQR)]	161.00 [158.00, 166.00]	161.00 [156.00,166.00]	0.273^c^
Weight [median (IQR)]	88.00 [72.00, 107.00]	79.00 [64.00,99.75]	0.001^a^ ^,^ ^c^
BMI [median (IQR)]	33.75 [27.78, 39.79]	30.86 [25.48,37.98]	0.003^a^ ^,^ ^c^
Tumor invasion (%) [median (IQR)]	36.00 [12.00, 57.50]	49.00 [18.50,67.00]	0.041^a^ ^,^ ^c^

a Statistically significant.

b Fisher exact test.

c Wilcoxon rank sum test.

**FIGURE 3:**
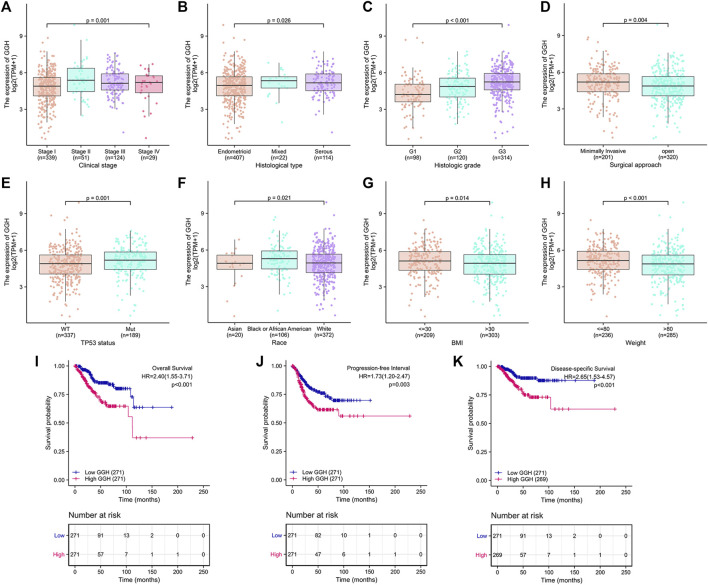
Associations between GGH expression level and various clinicopathological factors, including patient outcome, from TCGA. **(A–H)** GGH expression level (low vs. high) was significantly associated with clinical stage (*n* = 543 total cases, *p* = 0.001), histological type (*n* = 543, *p* = 0.026), histological grade (*n* = 532, *p* < 0.001), surgical approach (*n* = 521, *p* = 0.004), TP53 mutation status (*n* = 526, *p* = 0.001), race (*n* = 498, *p* = 0.021), BMI (*n* = 512, *p* = 0.014), and weight (*n* = 521, *p* < 0.001). **(I–K)** Kaplan-Meier analysis indicated poorer OS, PFI, and DSS among UCEC patients with high GGH mRNA expression. HR, hazard ratio; OS, overall survival; PFI, progression-free interval; DSS, disease-specific survival.

Additional analyses confirmed relationships between GGH expression and clinical stage, histological type, histological grade, surgical approach, TP53 mutation status, race, BMI, and weight (Figures 3A–H). Logistic regression analysis also revealed that high GGH expression was significantly associated with more advanced clinical stage [odds ratio (OR) = 1.90 for Stages II/III/IV vs. Stage I, *p* < 0.001], histological grade (OR = 3.00 for G3 vs. G1 and G2, *p* < 0.001), histological type (OR = 1.61 for Serous vs. Endometrioid, *p* = 0.026), and TP53 status (OR = 1.61 for Mutant vs. Wildtype, *p* < 0.001) (Table 2).

**TABLE 2:** Associations of GGH expression^a^ levels with clinicopathological characteristic according to logistic regression analysis.

Characteristics	Total (N)	Odds ratio (OR)	*p*-value
Clinical stage (Stage II & Stage III & Stage IV vs. Stage I)	543	1.90 (1.34–2.71)	<0.001
Histologic grade (G3 vs. G1 & G2)	532	3.00 (2.10–4.32)	<0.001
Residual tumor (R1 & R2 vs. R0)	410	1.32 (0.67–2.61)	0.42
Tumor invasion (%) (≥50 vs. <50)	470	1.23 (0.85–1.77)	0.274
Primary therapy outcome (CR vs. PD & SD & PR)	474	0.55 (0.28–1.08)	0.086
Histological type (Serous vs. Endometrioid)	521	1.61 (1.06–2.47)	0.026
TP53 status (Mutant vs. Wild-type)	526	1.86 (1.30–2.67)	<0.001
Surgical approach (Minimally Invasive vs. Open)	521	1.75 (1.23–2.51)	0.002
Menopause status (Post vs. Pre and Peri)	497	1.31 (0.74–2.36)	0.356
Radiation therapy (No vs. Yes)	518	0.94 (0.67–1.33)	0.74
Hormones therapy (No vs. Yes)	339	1.00 (0.53–1.89)	0.991
Diabetes (No vs. Yes)	442	0.99 (0.65–1.50)	0.945
Race (Asian and African American vs. White)	498	1.62 (1.08–2.45)	0.021
Age (≤60 vs. >60)	540	0.87 (0.62–1.23)	0.438
Weight (≤80 vs. >80)	521	1.89 (1.33–2.68)	<0.001
Height (≤160 vs. >160)	514	1.15 (0.81–1.63)	0.423
BMI (≤30 vs. >30)	512	1.69 (1.18–2.41)	0.004
TP53 status (Mutant vs. Wild-type)	526	1.86 (1.30–2.67)	<0.001

a Categorical dependent variable, greater, or less than the median expression level.

### Prognostic Value of GGH Expression in UCEC

**FIGURE 4:**
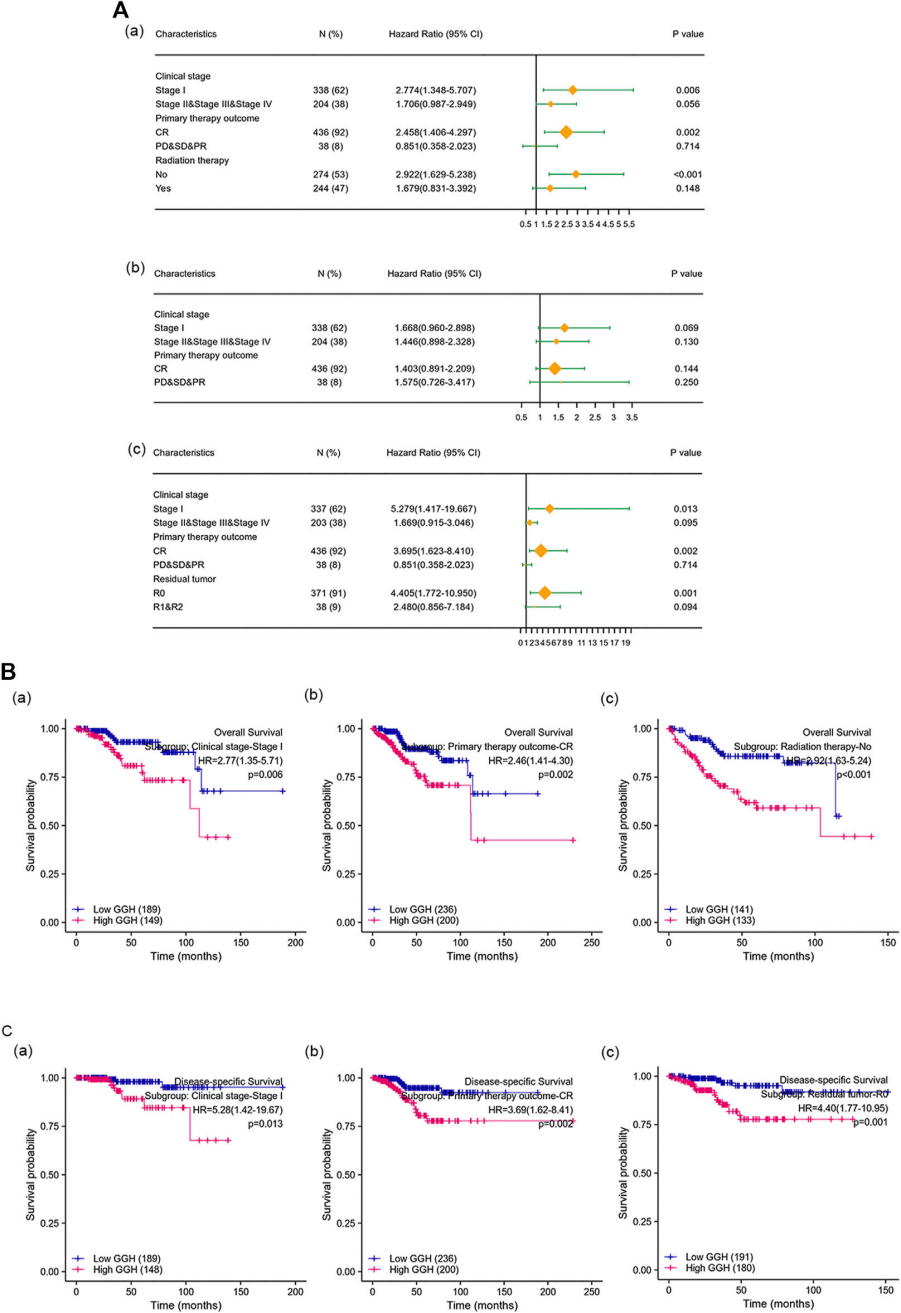
The prognostic value of GGH for survival prediction within specific clinicodemographic subgroups (chosen according to significant associations by multivariate Cox analysis). **(A)** Forest plots of the prognostic value in each subgroup for OS (a), PFI (b), and DSS (c). **(B–C)** Kaplan-Meier analysis of OS and DSS for each subgroup.

Kaplan-Meier analysis showed that higher GGH expression was associated with shorter OS [HR = 2.40 (1.55–3.71), *p* < 0.01], PFI [HR = 1.73 (1.20–2.47), *p* = 0.003], and DSS [HR = 2.65 (1.53–4.57), *p* < 0.001] (Figures 3I–K). Univariate analysis further showed that clinical stage (Stages II/III/IV vs. Stage I), histological grade (G3 vs. G1 & G2), residual tumor (R1 & R2 vs. R0), tumor invasion (%) (≥ 50 vs. < 50), primary therapy outcome [complete remission (CR) vs. PD/SD/PR], histological type (serous vs. endometrioid), TP53 status (Mutant vs. Wildtype), and GGH expression (high vs. low) were significantly correlated with OS, PFI, and DSS (Table 3). Moreover, age (>60 vs. ≤60) and radiation therapy (Yes vs. No) were also significantly associated with OS, and surgical approach (minimally invasive vs. open) was significantly associated with PFI (Table 3). Multivariate Cox analysis showed that clinical stage, primary therapy outcome, radiation therapy, and GGH expression were independently correlated with OS; clinical stage and primary therapy outcome were independently correlated with PFI; and clinical stage, primary therapy outcome, residual tumor, and GGH expression were independently correlated with DSS (Table 3). Thus, high GGH expression is a strong independent predictor of poor prognosis. To elucidate the mechanisms contributing to poor UCEC survival under elevated GGH expression, we first investigated the prognostic value of GGH for OS, PFI, and DSS prediction in each clinicodemographic subgroup showing significance by multivariate Cox analysis. The GGH expression level was a significant predictor of OS for the clinical Stage I subgroup, the primary therapeutic outcome CR subgroup, and the No radiation therapy subgroup. The GGH expression level was also a significant predictor of DSS for the clinical Stage I subgroup, the residual tumor R0 subgroup, and the CR subgroup, while high GGH expression had no prognostic value for PFI in any subgroup (Figure 4A). Kaplan-Meier analysis confirmed that high GGH expression was associated with worse OS in the clinical Stage I, CR, and No radiation subgroups (Figure 4B), and with worse DSS in the Stage I, CR, and R0 subgroups (Figure 4C). Collectively, these findings confirm that high GGH expression is strongly associated with poor prognosis among patients with UCEC.

**TABLE 3:** Associations of survival outcomes with clinicopathologic characteristics in TCGA patients by univariate and multivariate analyses.

Characteristics	Total (N)	Univariate analysis		Multivariate analysis	
		HR (95% CI)	*p*-value	HR (95% CI)	*p-*value
Overall survival					
Clinical stage (Stage II & Stage III & Stage IV vs. Stage I)	542	3.667 (2.377–5.657)	<0.001	3.071 (1.389–6.791)	0.006
Primary therapy outcome (CR vs. PD & SD & PR)	474	0.131 (0.079–0.218)	<0.001	0.256 (0.111–0.586)	0.001
Radiation therapy (Yes vs. No)	518	0.623 (0.402–0.964)	0.034	0.348 (0.178–0.679)	0.002
GGH (High vs. Low)	542	2.400 (1.553–3.709)	<0.001	2.442 (1.240–4.809)	0.01
Progression-free interval					
Clinical stage (Stage II & Stage III & Stage IV vs. Stage I)	542	2.675 (1.870–3.827)	<0.001	2.224 (1.179–4.194)	0.014
Primary therapy outcome (CR vs. PD & SD & PR)	474	0.120 (0.078–0.184)	<0.001	0.131 (0.066–0.262)	<0.001
Disease-specific survival					
Clinical stage (Stage II & Stage III & Stage IV vs. Stage I)	540	7.738 (4.102–14.596)	<0.001	5.030 (1.541–16.418)	0.007
Residual tumor (R1 & R2 vs. R0)	409	5.839 (3.145–10.841)	<0.001	2.889 (1.106–7.551)	0.03
Primary therapy outcome (CR vs. PD & SD & PR)	474	0.074 (0.042–0.131)	<0.001	0.161 (0.061–0.426)	<0.001
GGH (High vs. Low)	540	2.646 (1.533–4.566)	<0.001	3.167 (1.205–8.325)	0.019

### Construction of Nomograms for Patients With UCEC

**FIGURE 5:**
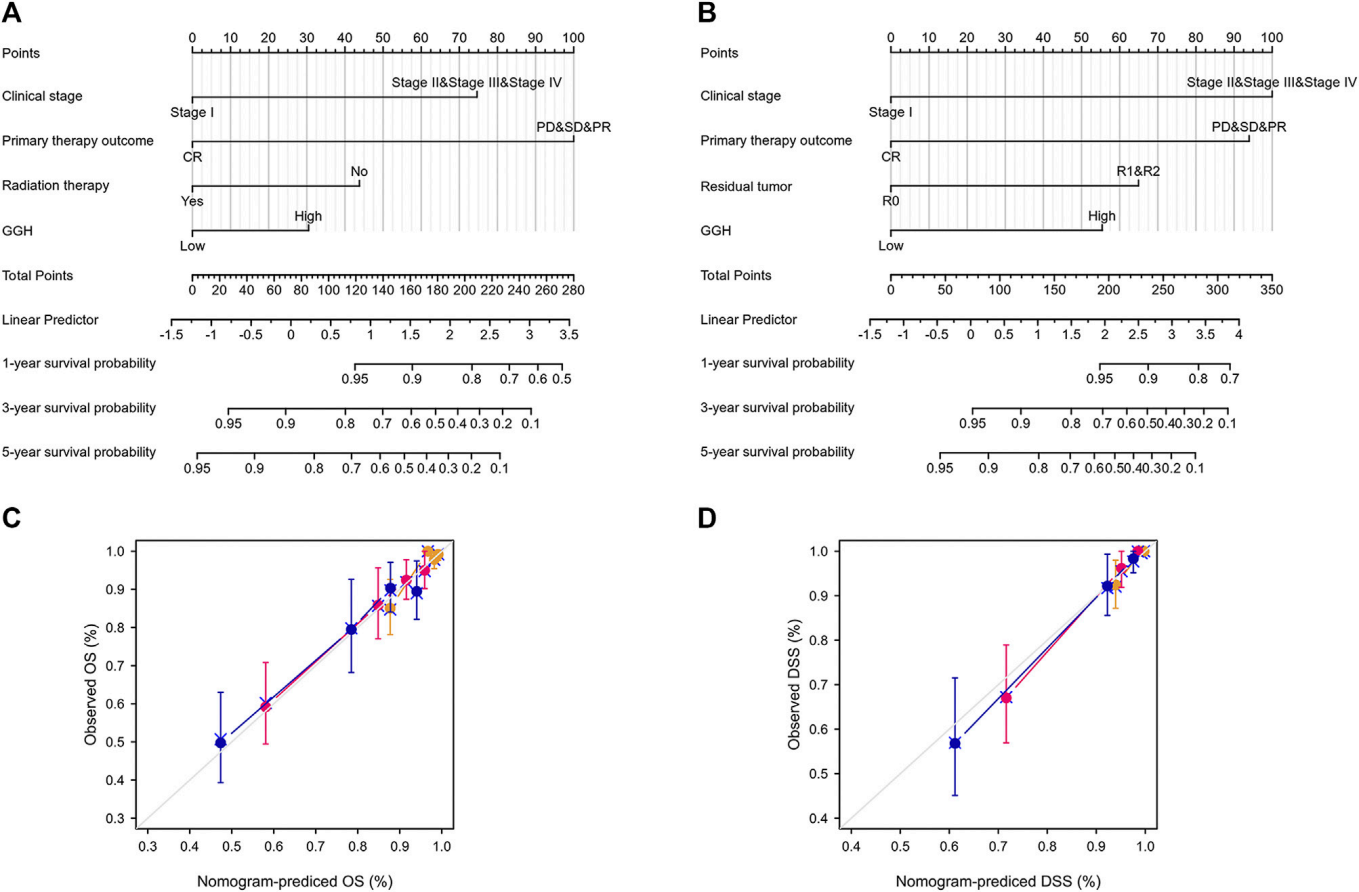
Construction and performance validation of GGH-based nomograms for UCEC patients. **(A,B)** Nomograms to predict the probability of 1-, 3-, and 5-years OS and DSS for UCEC patients. **(C,D)** Calibration plots of the sample nomograms for predicting OS and DSS probabilities showing the difference between true and predicted values.

Nomograms were constructed to integrate GGH expression and other prognostic factors demonstrated to be significantly predictive of OS and DSS by multivariate Cox analysis. In Figure 5, worse prognosis is represented by a higher total number of points on the nomogram. For OS, a UCEC patient in Stage II, III, or IV (75 points), achieving only PD, SD, or PR (100 points), receiving no radiation therapy (43 points), and with high GGH expression (31 points) would attain a total score of 249 points. The probability of l-year survival was determined by drawing a vertical line from the total point axis (at 249 in this example) downward to the outcome axis, which showed a 1-year survival probability of 64% (Figure 5A). The c-index for the nomogram was 0.789 with 1000 bootstrap replicates (95% CI: 0.759–0.819). Nomograms for DSS showed that a UCEC patient with Stage II, III, or IV (100 points), residual tumor index of R0 (94 points), achieving PD, SD, PR (65 points), and exhibiting high GGH expression (55 points) would attain a total point score of 314, for a 1-year survival probability of less than 70%. The c-index for the nomogram was 0.884 (95% CI: 0.861–0.907) (Figure 5B). The bias-corrected line in the calibration plot was close to the ideal curve (the 45-degree line) for OS and DSS, indicating good agreement between prediction and observation (Figures 5C,D).

### GGH-Related Genes and Their Functional Network

**FIGURE 6:**
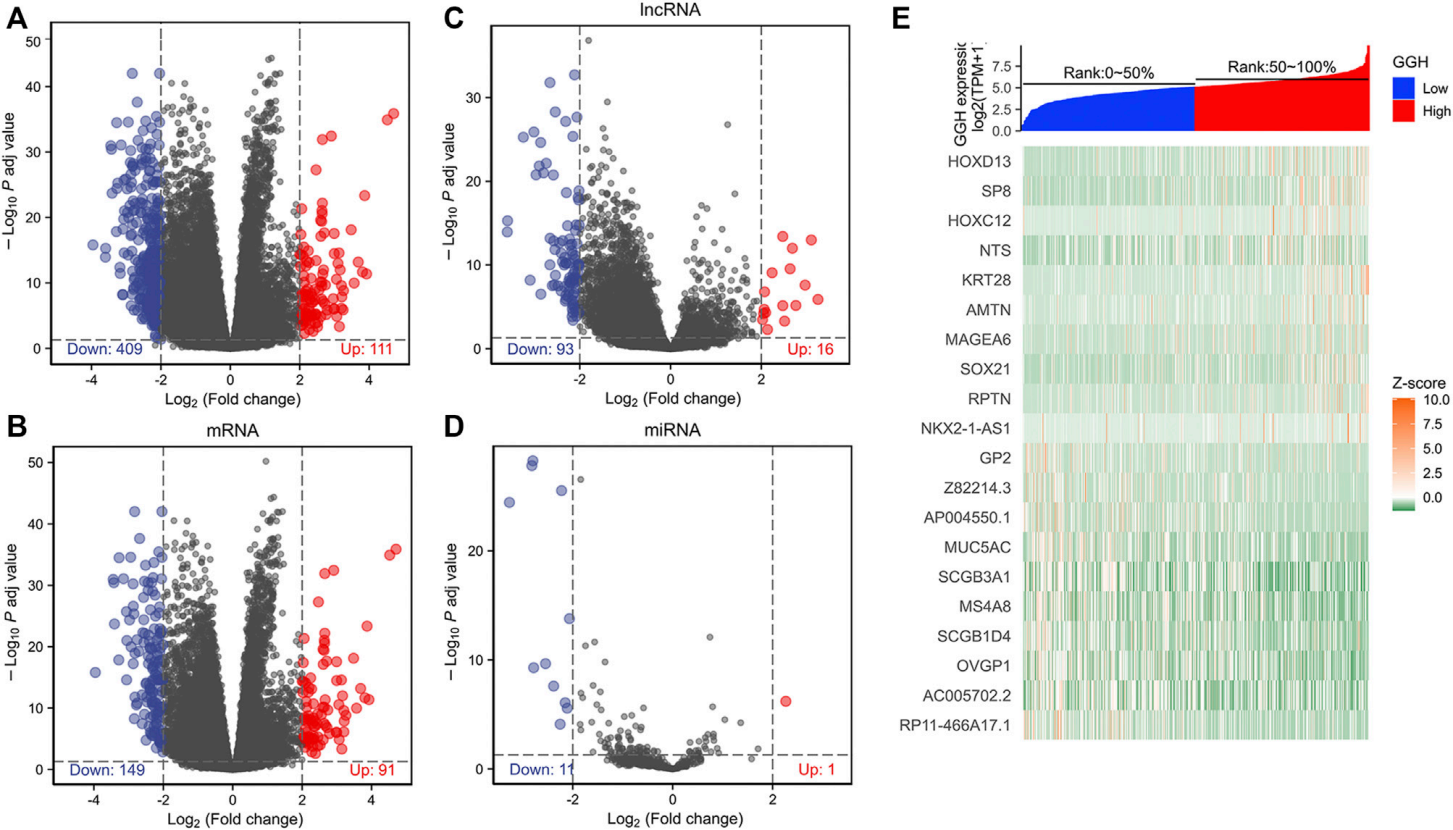
Differentially expressed genes between high and low GGH expression groups from TCGA. **(A–D)** Volcano plots of total DEGs, mRNAs, miRNAs, and lncRNAs. Red plots indicate upregulated genes, blue plots indicate downregulated genes, and the black plots show those with differential expression below the cutoff criteria. **(E)** Heatmap of the top 10 upregulated genes and top 10 downregulated genes between high and low GGH expression subgroups.

To investigate the GGH-related mechanisms in UCEC, we identified the genes that were differentially expressed between patients with high and low GGH expression and then analyzed their function and signaling pathways as well as the PPI of GGH-related genes. A total of 520 DEGs (111 upregulated and 409 downregulated) were identified between high and low GGH expression groups, including 240 mRNAs (91 upregulated and 149 downregulated), 12 miRNAs (1 upregulated and 11 downregulated), and 109 lncRNAs (16 upregulated and 93 downregulated) (Figures 6A–D). The expression levels of the top 10 upregulated and downregulated DEGs are illustrated by the heat map in Figure 6E.

**FIGURE 7:**
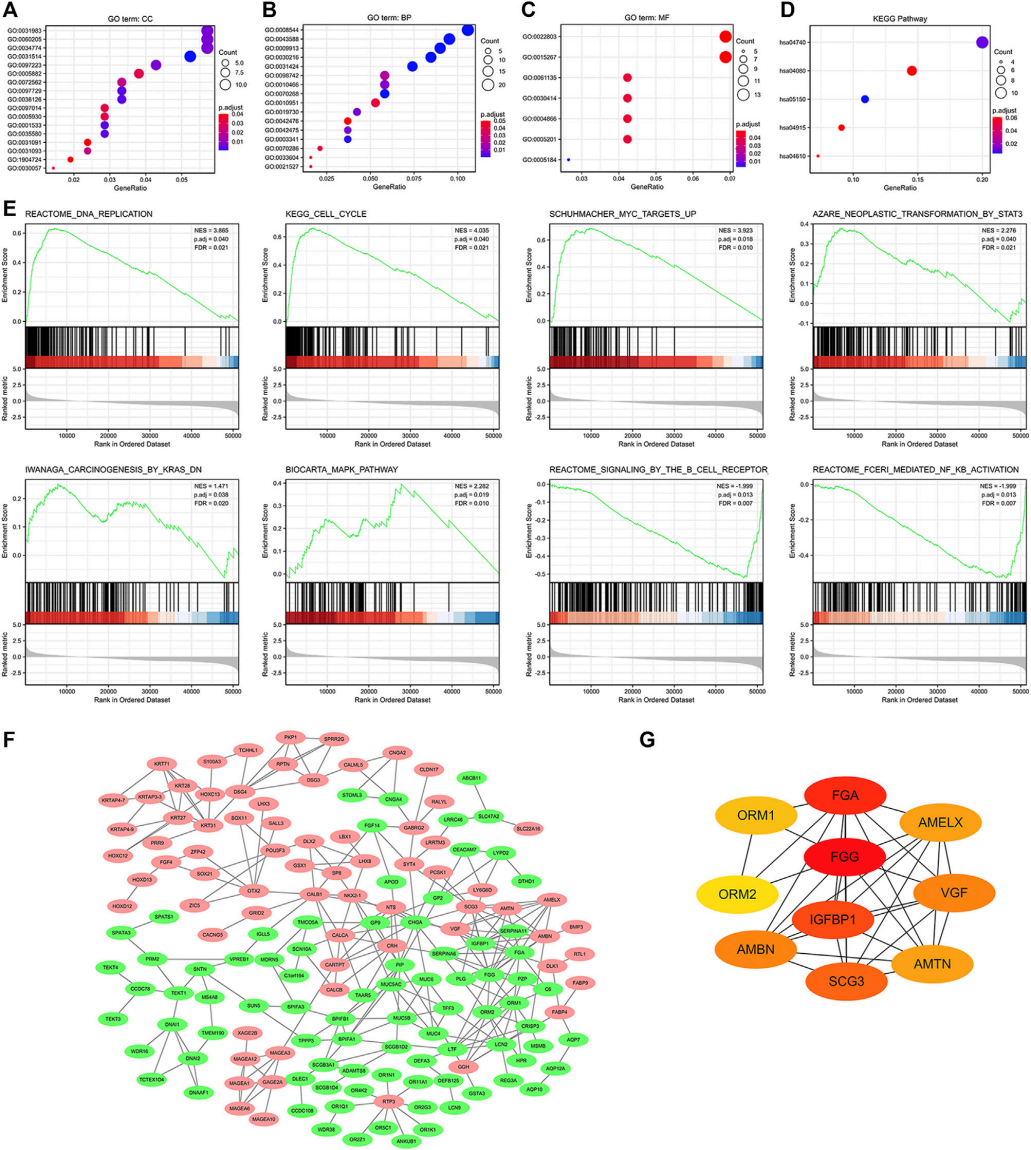
Functional enrichment analysis of 521 DEGs between high and low GGH expression subgroups of patients with UCEC from TCGA. **(A–C)** Enriched GO terms in the “cellular component” category **(A)**, “biological process” category **(B)**, and “molecular function” category **(C)**. **(D)** KEGG classification map of DEGs. The *x*-axis represents the proportion of DEGs, and the *y*-axis represents the individual GO or KEGG terms. The different colors indicate adjusted *p*-values, and the different dot sizes represent the number of DEGs associated with each term. **(E)** Enrichment plots from gene set enrichment analysis (GSEA). Several pathways were differentially enriched in GGH-related genes, including “FC-gamma receptor (FCγR),” “MHC class II antigen presentation,” “oncogenesis by met,” “DNA replication,” “ubiquitin-mediated proteolysis,” “MAPK pathway,” “cell cycle,” and “endocrine therapy resistance.” ES, enrichment score; NES, normalized ES; *p*.adj, adjust *p-*value; FDR. **(F)** Visualized protein–protein interaction enrichment analysis of DEGs. Red plots represent upregulated DEGs and green plots represent downregulated DEGs. **(G)** Top 10 genes from the PPI network calculated by the MCC method using Cytohubba. Yellow indicates low and red indicates high scores.

We further established the lncRNA-miRNA-mRNA regulation network containing 6 lncRNAs, 4 miRNAs, and 65 mRNAs by using the Cytoscape software (Supplementary Figure S2). Results of GO functional enrichment analysis showed that these DEGs engaged in several BPs, CCs, and MFs. DEGs were linked primarily to “motile cilium” (GO: 0031514), “intermediate filament” (GO: 0005882), and “desmosome” (GO: 0030057) (Figure 7A). In the biological process category, DEGs were mainly enriched in “keratinocyte differentiation” (GO: 0030216), “cilium movement” (GO: 0003341), and “antimicrobial humoral response” (GO: 0019730) genes (Figure 7B), thus suggesting a link between aberrant GGH expression and cell movement. The two major molecular functions for these genes were “transcription regulation by the extracellular matrix structural constituent” (GO: 0005201) and “peptidase inhibitor activity” (GO: 0030414) (Figure 7C). Additionally, KEGG analysis showed that GGH-interactive genes also included those related to “*Staphylococcus aureus* infection” and “olfactory transduction” (Figure 7D). The GGH-related signaling pathways involved in UCEC were selected based on NES values. The tumorigenesis-associated pathways “oncogenesis by met,” “ubiquitin-mediated proteolysis,” “cell cycle,” “endocrine therapy resistance,” “DNA replication,” “MAPK pathway,” “MHC class II antigen presentation,” and “FC-gamma receptor-dependent phagocytosis” were significantly enriched in GGH-regulated genes among patients with UCEC with high GGH expression (Figure 7E). A PPI network including GGH and co-expressed DEGs was then constructed (Figure 7F). The top ten genes in this PPI network were *FGG*, *FGA*, *IGFBP1*, *SCG3*, *AMBN*, *VGF*, *AMELX*, *AMTN*, *ORM1*, and *ORM2* (Figure 7G).

### Correlations Between GGH Expression and Immune Cell Infiltration

**FIGURE 8:**
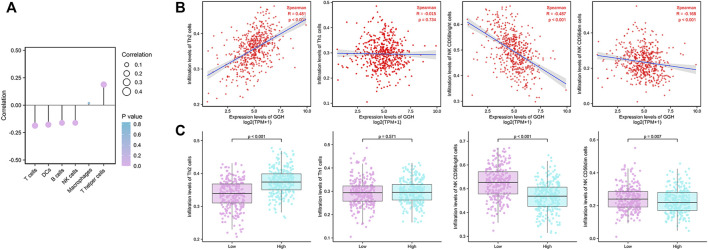
Associations between GGH mRNA expression level and infiltration of various immune cell types into the UCEC tumor microenvironment. **(A)** Correlations between GGH expression level and the relative abundances of the indicated immune cells. Dot size indicates the absolute Spearman correlation coefficient (R), and the color gradation from blue to pink indicates low to high *p*-value. **(B)** Correlations between the relative enrichment scores of individual immune cell type genes and GGH expression level. **(C)** Comparisons of immune cell infiltration between high and low GGH expression groups.

Finally, we analyzed the associations between GGH expression level and the infiltration of various immune cell types into the UCEC tumor microenvironment. GGH expression was negatively correlated with the infiltration of T cells, dendritic cells, B cells, and natural killer (NK) cells and positively correlated with the infiltration of T helper (Th) cells (Figure 8A). Furthermore, GGH expression was significantly correlated with greater Th2 cell infiltration (*r* = 0.481, *p* < 0.001) but lower NK CD56bright cell infiltration (*r* = −0.487, *p* < 0.001) (Figure 8B). Compared to tissues with low GGH expression, Th2 cell infiltration was significantly higher while NK CD56bright cell infiltration was significantly lower in tissues with high GGH expression (Figure 8C). We also used other algorithms in the TIMER2.0 (Li et al., 2020) database to verify the relationship between GGH expression and immune cell infiltration (Supplementary Figure S3).

## Discussion

Folic acid is the synthetic form of folate and one of the most common nutrients for women. Excessive intake of folic acid has been reported to increase the risk of cancers including type II endometrial cancer (Kim, 2008; Figueiredo et al., 2009; Uccella et al., 2011). Therefore, the relative expression status of key enzymes involved in folate metabolism is worthy of study in gynecological tumors. GGH is a lysosomal enzyme that catalyzes the formation of monoglutamyl folate, which in turn influences DNA synthesis (Kim, 2020). It is highly expressed in the human kidneys, liver, fetal tissue, and placenta, whereas its expression in the spleen, lungs, small intestine, and peripheral blood leukocytes is relatively low (Yin et al., 2003). Overexpression of GGH has been implicated in many diseases, and high expression and abnormal activity have been detected in multiple cancer cell lines and tumor tissues (Schneider and Ryan, 2006). In this study, we demonstrated that GGH is aberrantly overexpressed in UCEC and that higher overexpression is associated with lower survival probability, possibly through direct or DEG-mediated effects on cell proliferation and immune responses among other pathways. We conclude that GGH expression is a reliable prognostic indicator for UCEC. In addition, GGH and associated molecular pathways identified by our bioinformatics analyses may be novel therapeutic targets for UCEC treatment.

Previous studies have reported higher GGH expression levels in urothelial, invasive breast, ERG-negative prostate, gallbladder, and gastric cancers compared to corresponding control tissues (Pollard et al., 2009; Silva et al., 2013; Wang et al., 2014; Zali et al., 2019; Muralidharan et al., 2020), which is consistent with our pancancer analysis of TCGA data showing higher GGH expression in numerous cancers including other gynecologic cancers. This elevated expression was observed at both protein and mRNA levels in UCEC patients. ROC analysis also indicated that GGH expression accurately distinguished UCEC tumors from nontumor tissues. Thus, GGH is likely to be critical for certain basic tumorigenic processes, a speculation confirmed by our subsequent bioinformatics analysis. Further, GGH expression was associated with greater tumor progression and poorer outcome. Similarly, high GGH expression was significantly associated with histological tumor grade in breast cancer (BRE III, *p* < 0.001) (Shubbar et al., 2013). In gastric cancer patients as well, high GGH mRNA expression was significantly associated with histological type and vascular invasion (Maezawa et al., 2020). In our study, greater GGH expression was associated with higher clinical stage, higher histological grade, and mixed histological type. In addition, GGH expression was significantly higher in UCEC patients receiving minimally invasive surgery, those carrying the TP53 mutation, black or African-American patients, and patients with BMI ≤30 or weight ≤80 kg, all of which are associated with lower survival rates in other cancers (Palumbo et al., 2002; Melamed et al., 2018; Donehower et al., 2019; Chasov et al., 2021).

Several previous studies have reported GGH expression as a prognostic indicator for some tumors, but there have also been inconsistencies. For instance, the 10-years recurrence-free survival rate of ERG-negative prostate cancer patients with high GGH expression levels was significantly higher than that of patients with low GGH expression levels (Melling et al., 2017). In contrast, the 5-years OS rate of gastric cancer patients with high GGH mRNA expression level was significantly lower than that of patients with low GGH expression (Maezawa et al., 2020). There was also a significant difference in 8-years DSS between GGH-expressing and GGH-negative invasive breast cancer patients, and the risk of death was 2.7 times greater in the high GGH expression group (Shubbar et al., 2013). In the present study, survival analysis indicated that high GGH expression was strongly associated with shorter OS, PFI, and DSS. Our results also revealed that clinical stage and primary therapy outcome are independent prognostic factors for UCEC, in accord with previous studies on other cancer types (Morice et al., 2016). Hence, GGH appears to be a reliable prognostic marker. Indeed, the c-index and highly fitted calibration plots demonstrated that nomograms for OS and DSS including GGH accurately predicted UCEC patient survival. Furthermore, high GGH expression strongly predicted shorter OS and DSS in specific clinical subgroups. Collectively, these findings suggest that GGH overexpression contributes directly or indirectly to biological processes underlying tumor aggression and hence poor outcome. Subsequent bioinformatics analyses provided further support for this notion.

We identified 520 DEGs between high and low GGH expression groups from the TCGA RNA sequencing data and constructed a lncRNA-miRNA-mRNA regulation network, containing 6 lncRNAs, 4 miRNAs, and 65 mRNAs. GO function and KEGG pathway enrichment analyses showed that these DEGs were mainly related to cell movement and infection immunity. This finding is in accordance with a previous study reporting that decreased GGH expression reduced the migration of an esophageal squamous cell carcinoma cell line (Peng et al., 2019). Results of GSEA analysis also revealed the associations of GGH with other cancer-related molecular pathways, including DNA replication, cell cycle, MAPK, KRAS, STAT3, and B cell receptor. Previous studies have shown that activation of MAPK, KRAS, or STAT3 pathways alter cell proliferation, apoptosis, and other biological behaviors that influence the occurrence and progression of UCEC (Fathi et al., 2018; Li et al., 2018; Sideris et al., 2019). It was also reported that changes in the GGH-dependent regulation of folate concentration affect intracellular mitochondrial metabolism, gene expression, DNA methylation, and DNA repair efficiency (Kim et al., 2015; Kim, 2020). Higher expression of GGH can accelerate the conversion of polyglutamyl folate to the monoglutamyl form and reduce total folate levels. The monoglutamate form can stimulate glioma cell proliferation by activating MAPK and PI3K/AKT pathways (Schneider and Ryan, 2006; Oleinik et al., 2014; Robert and Sontheimer, 2014). In addition, the hub genes identified in the associated PPI network regulate cell growth, cell proliferation, cell apoptosis, DNA methylation, and inflammation (de Maat et al., 2010; Ando et al., 2017; Marwitz et al., 2017; Wang et al., 2020; Zhu et al., 2020). Taken together, these results indicate that GGH may drive UCEC tumor progression by engaging multiple interacting molecular pathways involved in cell proliferation, migration, metabolism, apoptosis, and immune responses.

The types of immune cells infiltrating into the tumor microenvironment are strongly indicative of tumor progression status. In the current study, we found significant associations between GGH expression level and the infiltration of NK, dendritic, B, T, and Th cells. Higher expression was associated with greater infiltration of Th2 cells and lower infiltration of NK CD56bright cells. The main effectors of Th1 cells are interleukin (IL)-2 and interferon (IFN)-γ, which can indirectly or directly promote and maintain the proliferation and activation of T cells, induce and enhance the anti-tumor activity of NK cells, and inhibit the division and proliferation of tumor cells (Thakur et al., 2012). Alternatively, Th2 cells can contribute to tumor immune escape through production of IL-10 and IL-4, both of which inhibit the response of Th1 cell effectors, the verification response of inflammatory cells, antigen promotion, and T cell proliferation (Thakur et al., 2012). The increased infiltration of Th2 cells under GGH overexpression with no significant effect on Th1 cell infiltration may lead to a Th1/Th2 imbalance that allows tumor cells to resist immune attack. Moreover, NK CD56dim cells have been shown to kill tumor cells, whereas NK CD56bright cells are generally thought to play an “accomplice” role in tumor formation (Caligiuri, 2008). However, recent studies have found that NK CD56bright cells have greater tumor-killing capacity than NK CD56dim cells (Poznanski et al., 2018). Thus, the lower NK CD56bright cell infiltration under elevated GGH expression may also allow for tumor progression unchecked by immune attack.

Although our study demonstrates a pathogenic function for GGH overexpression in UCEC, there were several limitations. First, we verified overexpression at the protein level using a limited number of patient samples. Second, DEGs were identified from expression databases and none were confirmed by PCR or Western blotting. Third, the precise functions of these DEGs in UCEC are largely unexplored.

## Conclusion

Expression of GGH is elevated in UCEC, and higher expression is associated with more severe clinicopathological characteristics and poorer prognosis. Overexpression of GGH may be associated with dysregulation of multiple cancer-related gene pathways, including those involved in cell cycle regulation, cell motility, MAPK, STAT3, and KRAS signaling, and immune responses. Further, GGH overexpression may reduce tumor immune attack by altering the immunocyte infiltration profile. This study identifies GGH as a potentially useful biomarker for detection and prognosis of UCEC. Furthermore, GGH and associated molecular pathways may be effective therapeutic targets for UCEC treatment.

## Data Availability

The datasets presented in this study can be found in online repositories. The names of the repository/repositories and accession number(s) can be found in the article/Supplementary Material.
