# Supplementary figures and images for: Elevated Expression of Gamma-Glutamyl Hydrolase Is Associated With Poor Prognosis and Altered Immune Signature in Uterine Corpus Endometrial Carcinoma
Source: Front Genet. 2022 Jan 10;12:764194. doi: 10.3389/fgene.2021.764194 (PMC8785095; doi:10.3389/fgene.2021.764194)

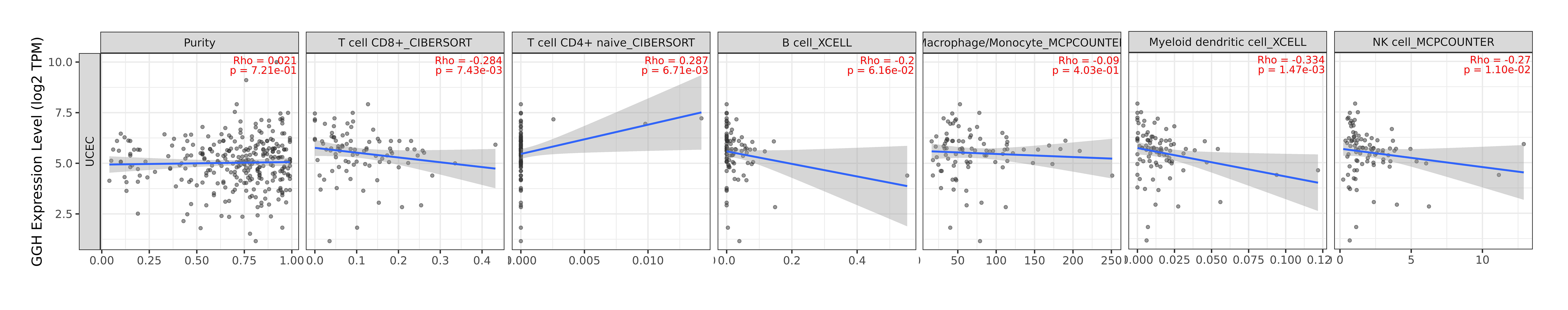

Supplement: Supplementary file 1 [file Image3.JPEG]

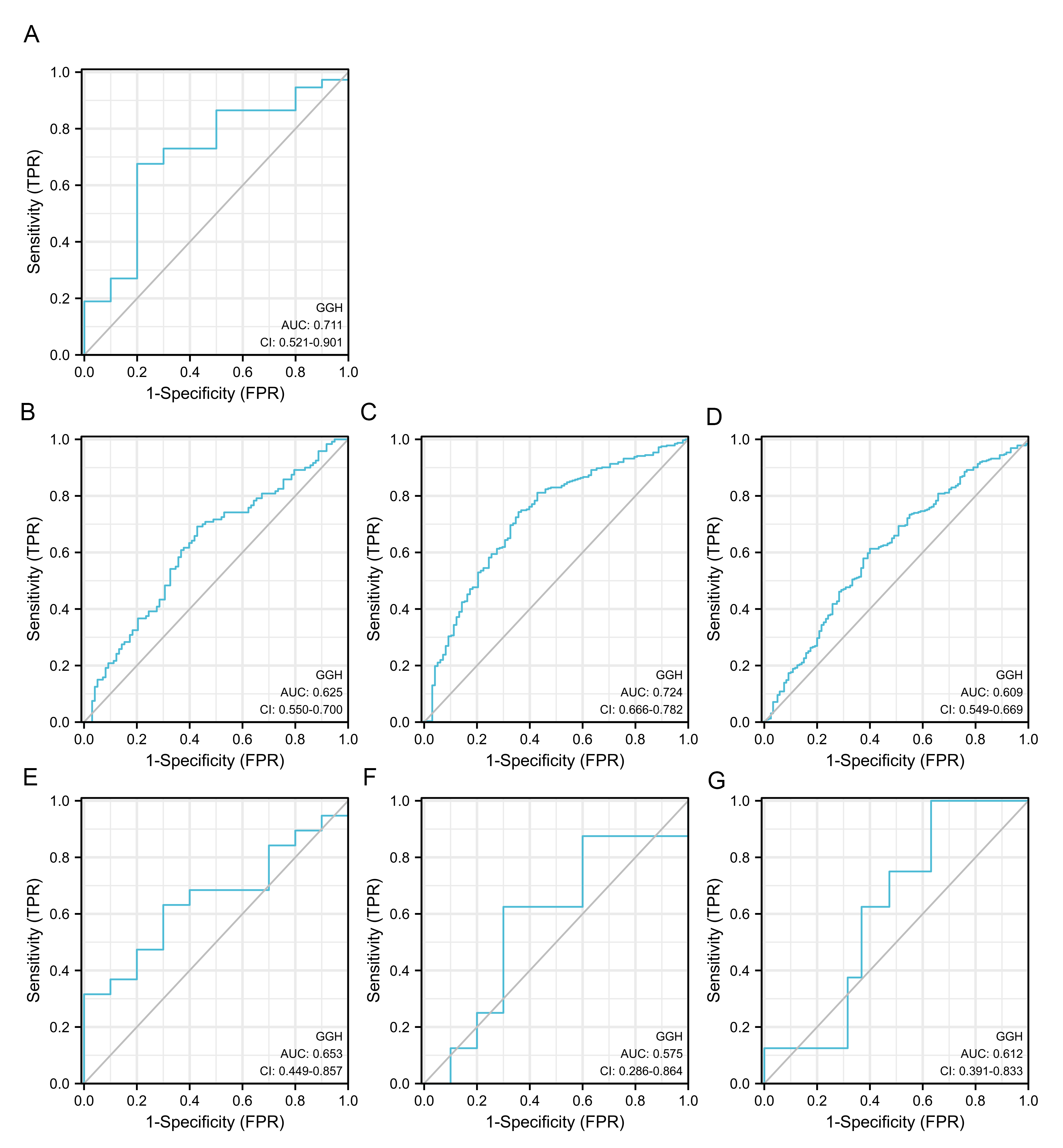

Supplement: Supplementary file 2 [file Image1.JPEG]

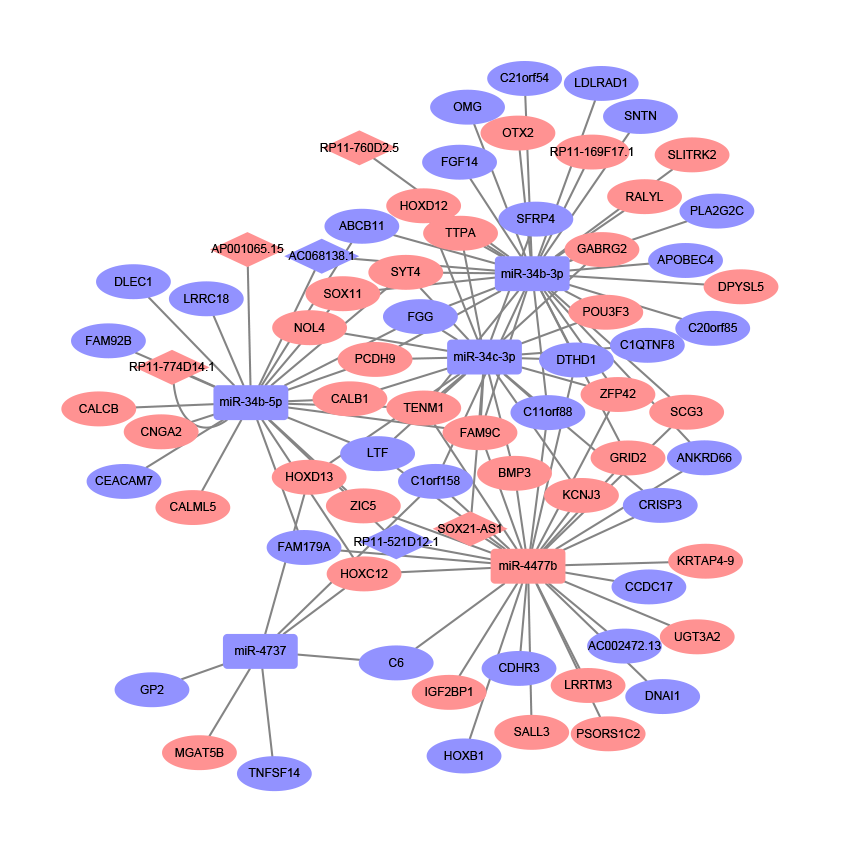

Supplement: Supplementary file 3 [file Image2.TIF]
